# Exploring the Views of Dermatologists, General Practitioners, and Melanographers on the Use of AI Tools in the Context of Good Decision-Making When Detecting Melanoma: Qualitative Interview Study

**DOI:** 10.2196/63923

**Published:** 2025-03-24

**Authors:** Brad Partridge, Nicole Gillespie, H Peter Soyer, Victoria Mar, Monika Janda

**Affiliations:** 1 Centre for Health Services Research University of Queensland Brisbane Australia; 2 Melbourne Business School University of Melbourne Melbourne Australia; 3 School of Business University of Queensland Brisbane Australia; 4 Frazer Institute, Dermatology Research Centre University of Queensland Brisbane Australia; 5 Victorian Melanoma Service Alfred Health Melbourne Australia; 6 School of Public Health and Preventive Medicine Monash University Melbourne Australia

**Keywords:** artificial intelligence, melanoma, skin cancer, decision-making, decision support, qualitative, attitudes, dermatologists, general practitioners, melanographers, Australia, New Zealand

## Abstract

**Background:**

Evidence that artificial intelligence (AI) may improve melanoma detection has led to calls for increased human-AI collaboration in clinical workflows. However, AI-based support may entail a wide range of specific functions for AI. To appropriately integrate AI into decision-making processes, it is crucial to understand the precise role that clinicians see AI playing within their clinical deliberations.

**Objective:**

This study aims to provide an in-depth understanding of how a range of clinicians involved in melanoma screening and diagnosis conceptualize the role of AI within their decision-making and what these conceptualizations mean for good decision-making.

**Methods:**

This qualitative exploration used in-depth individual interviews with 30 clinicians, predominantly from Australia and New Zealand (n=26, 87%), who engaged in melanoma detection (n=17, 57% dermatologists; n=6, 20% general practitioners with an interest in skin cancer; and n=7, 23% melanographers). The vast majority of the sample (n=25, 83%) had interacted with or used 2D or 3D skin imaging technologies with AI tools for screening or diagnosis of melanoma, either as part of testing through clinical AI reader studies or within their clinical work.

**Results:**

We constructed the following 5 themes to describe how participants conceptualized the role of AI within decision-making when it comes to melanoma detection: theme 1 (integrative theme)—the importance of good clinical judgment; theme 2—AI as just one tool among many; theme 3—AI as an adjunct after a clinician’s decision; theme 4—AI as a second opinion for unresolved decisions; theme 5—AI as an expert guide before decision-making. Participants articulated a major conundrum—AI may benefit inexperienced clinicians when conceptualized as an “expert guide,” but overreliance, deskilling, and a failure to recognize AI errors may mean only experienced clinicians should use AI “as a tool.” However, experienced clinicians typically relied on their own clinical judgment, and some could be wary of allowing AI to “influence” their deliberations. The benefit of AI was often to reassure decisions once they had been reached by conceptualizing AI as a kind of “checker,” “validator,” or in a small number of equivocal cases, as a genuine “second opinion.” This raised questions about the extent to which experienced clinicians truly seek to “collaborate” with AI or use it to inform decisions.

**Conclusions:**

Clinicians conceptualized AI support in an array of disparate ways that have implications for how AI should be incorporated into clinical workflows. A priority for clinicians is the conservation of good clinical acumen, and our study encourages a more focused engagement with users about the precise way to incorporate AI into the clinical decision-making process for melanoma detection.

## Introduction

### Background

Timely access to accurate, cost-effective melanoma screening and diagnosis is an ongoing area of health care priority, particularly given that early treatment of melanoma is associated with the most favorable patient outcomes. The current detection paradigm relies heavily on clinician examination assisted by dermoscopy; therefore, accuracy is variable depending on the clinician’s level of experience and their personal risk threshold for performing a biopsy. Integrating artificial intelligence (AI) with 2D or 3D skin imaging technologies into clinical workflows and decision-making processes may improve melanoma detection in a number of ways. A growing number of studies indicate that, under experimental conditions, AI can correctly identify images of malignant lesions with a level of diagnostic accuracy that exceeds, or is at least comparable to, expert dermatologists [1-4]. AI may help to identify new or changing lesions in patients at high risk when lesion-specific or full-body images are taken sequentially or help to triage patients by identifying suspicious lesions that require more focused clinical assessment by dermatologists [5,6]. While regulatory approval and the integration of AI tools for melanoma diagnosis is not yet a widespread part of dermatological practice, some AI tools are being used in public and private health care settings in several countries to triage patients by identifying suspicious lesions that require more focused clinical assessment by dermatologists. For example, the Deep Ensemble for Recognition of Malignancy (DERM) AI device is approved for use in the United Kingdom and designed to be used for “screening, triage, and assessment of skin lesions” as well as to provide a “suggested diagnosis and referral recommendation” [6]. Using AI for these purposes may improve the efficiency of melanoma detection and increase population access to dermatological assessment, particularly as improvements in the precision of imaging technology and convolutional neural networks allow for more machine autonomy in decision-making, thereby changing or creating new clinical paradigms in melanoma detection.

Calls for human-AI collaboration in melanoma detection are based on the view that “AI-based support of clinical decision-making improves diagnostic accuracy over that of either AI or physicians alone” [7], and the dominant narrative is that AI should ideally be integrated into clinical workflows in a way that “assists” and “supports” clinical decision-making about skin cancer detection [1-3]. However, there is no consensus on how this integration ought to occur nor how or where in the process AI should be used by clinicians to make better decisions. For example, the way AI output is used and regarded may depend on whether it is referred to before or after the clinicians have made their own assessment. Furthermore, the use of AI as part of melanoma screening and diagnosis also strongly depends on clinicians’ acceptance of the technology. Therefore, understanding clinician views regarding the incorporation of AI into decision-making is crucial for developing appropriate clinical workflows (eg, a widespread melanoma screening program). To date, a small number of surveys with dermatologists and general practitioners (GPs) across several countries have provided broad level snapshots of overall inclinations toward the use of AI (Al-Ali et al [8], Nelson et al [9], Patrzyk et al [10], Polesie et al [11], Scheetz et al [12], Shen et al [13], Wei et al [14], and Samaran et al [15]). These surveys have generally reported favorable attitudes toward the potential for AI to positively impact dermatological practice in the future [11] as well as a perception that using AI may improve the efficiency of melanoma detection as the precision of imaging technology improves [12]. Studies using qualitative methods, such as focus groups, have allowed for more in-depth descriptions of clinician views on the potential benefits, barriers, and preconditions of using AI for skin cancer detection [16] in ways that elicit more detailed information about the reasoning and beliefs underlying clinician attitudes. Currently, this level of empirical detail is scarce when it comes to understanding the precise role that clinicians see AI playing within their clinical deliberations and how they think AI output ought to be used to inform their own decisions so that any potential benefits of AI can be wholly realized.

The extent to which AI is seen as helpful for making good decisions outside of experimental settings may vary markedly according to the clinical setting, user, and purpose [17,18]. Therefore, a more in-depth investigation of how clinicians conceptualize the specific way AI may be used within their actual decision-making process is needed, as is a more direct assessment that seeks to learn from the actual experiences clinicians may have already had with AI in dermatology. This does not only apply to dermatologists. In countries with high prevalence rates of melanoma, such as Australia and New Zealand [19], skin cancer diagnosis is conducted by dermatologists and GPs, (including those with a special interest in skin cancer), and screening may be conducted by other clinicians such as melanographers (skin imaging technicians who often have a background in nursing).

### Objectives

In recognizing the importance of this practice-driven approach, this study uses in-depth individual interviews with a range of clinicians involved in melanoma detection (dermatologists, GPs, and melanographers) to understand how they conceptualize and view the role of AI within their decision-making and what these conceptualizations mean for good decision-making when detecting melanoma. By drawing on the experiences that clinicians have already had with AI where possible, this understanding will help clinical groups, policy makers, and AI developers to respond to the needs of clinicians involved in the detection of melanoma when it comes to AI use.

## Methods

### Participants and Recruitment

This qualitative study used in-depth individual interviews to explore clinicians’ experiences with and views about using AI to detect melanoma. Melanoma is detected by either (1) screening asymptomatic patients for suspicious lesions and identifying new or changing lesions or (2) correctly diagnosing malignant lesions that the patient seeks advice about. We recruited a cohort of clinicians who regularly conduct skin cancer examinations, including dermatologists, GPs with an interest in skin cancer, and melanographers. We sought participants who had familiarity with AI use in the clinical setting or as part of research (but did not exclude those without experience with AI). Participants were recruited through purposive sampling from the authorship group’s network of (1) scientific contacts in melanoma research in Australia and New Zealand (including the Australian Centre of Excellence in Melanoma Imaging and Diagnosis, currently one of the largest melanoma cohort studies worldwide using 3D total body imaging for melanoma early detection) and (2) professional clinical contacts in Australia and New Zealand including GPs, melanographers, and fellows and trainees of the Australasian College of Dermatologists (the peak professional body for dermatologists in Australia and New Zealand). An initial group of potential participants was contacted via email, and additional prospective interviewees were contacted on the recommendation of participants themselves through “snowball” recruitment.

The final sample comprised 30 participants: 17 (57%) dermatologists, 6 (20%) GPs with a special interest in skin cancer, and 7 (23%) melanographers (refer to Table 1 for further sample demographics). A total of 26 (87%) participants were from Australia and New Zealand; 4 (13%) participants from Chile, Greece, the United States, and the United Kingdom were recruited through snowball sampling. The vast majority of the sample (n=25, 83%) had interacted with or used 2D or 3D skin imaging technologies with AI tools for screening or diagnosis of melanoma, either as part of experimental testing (or through clinical AI reader studies) or within their clinical work. Dermatologists and GPs in the sample collectively had experience with AI tools for predicting likely diagnosis, detecting lesion change, and screening. The melanographers in the sample had clinical experience with AI tools to screen lesions for melanoma (ie, identifying suspicious lesions in need of referral to a dermatologist). The most commonly nominated AI tools used by participants were part of imaging platforms provided by FotoFinder (FotoFinder systems, Inc), DermEngine (MetaOptima), Canfield (as part of 3D total body photography; Canfield Scientific Inc), and MoleMap.

**Table 1:** Demographics.

		Dermatologists (n=17)	General practitioners (n=6)	Melanographers (n=7)	Total sample (n=30)
**Gender, n (%)**					
	Men	10 (59)	4 (66)	0 (0)	14 (47)
	Women	7 (41)	2 (33)	7 (100)	16 (53)
Age (y), mean (SD; range)		44.5 (7.7; 32-60)	52.1 (12.2; 33-72)	34.7 (10.3; 26-49)	43.7 (6.4; 32-72)
Experience (y), mean (SD; range)		14.1 (7.7; 1-30)	13.8 (5.6; 5-20)	4.6 (4.9; 1-15)	11.4 (2.5; 1-30)
Experience with artificial intelligence, n (%)		14 (82)	4 (66)	7 (100)	25 (83)

### Data Collection and Analysis

All interviews were conducted by the first author (BP) via a video link between October 2023 and February 2024, with 2 (7%) of the 30 participants providing written input instead of an interview. The average duration of interviews was approximately 45 minutes, and interviews were digitally recorded with the participant’s consent and transcribed. A semistructured interview schedule developed by the research team was used to guide discussion on several areas, including experiences with skin imaging technologies and decision-making with AI tools, expectations for the use of AI as part of decision-making, clinician trust, and potential barriers and enablers of AI use in various clinical workflows.

Using a critical realist stance, we conducted a thematic analysis of transcripts by drawing on the template analysis approach to structure data coding [20,21]. BP read through all interview transcripts for familiarization. BP and NG then started preliminary coding of the data (using a process familiar across all forms of thematic analysis whereby the researcher identifies and “labels” text that may contribute to understanding of the topic; this is described by King et al [21]) by (1) reading through the first 6 interview transcripts together, (2) inductively coding these transcripts for meaning independently, and (3) collaboratively discussing shared interpretations and impressions where diverging views were settled via open discussion. We chose not to make use of a priori themes to guide coding and favored an inductive approach to the development of initial themes by identifying clusters of shared meaning across interviews. Early in the process of clustering, we identified how conceptualizations of AI were often linked to the way participants talked about “positioning” AI in the decision-making process, and this helped to formulate initial themes. As described in the study by King et al [21], this clustering also allows the development of potential “integrative themes,” that is, themes that permeate other clusters of meaning. We generated the integrative theme of “the importance of good clinical judgement” given the way it infused how AI was conceptualized throughout. The first iteration of a coding structure, with initial themes related to good clinical decision-making and the use of AI, was constructed and then applied to the whole dataset, undergoing several refinements, where necessary, to identify how meaningfully it captured the data. At its core, refinement of themes entails repeatedly going back to interview transcripts and “testing” how well the thematic descriptions capture meaning. Here, refinement typically entailed adjusting the wording of themes for clarity in order to ensure precise capture of the data. This process ensured continuous engagement with the data. Construction of themes was then solidified with all authors contributing to the final data interpretation.

### Ethical Considerations

This study was approved as low or negligible risk research by the University of Queensland Human Research Ethics Committee (2023/HE001714). All participants provided informed consent to participate and had the ability to opt out. Participants were not provided with compensation or incentives to participate. Data have been deidentified.

## Results

### Overview

To describe how participants viewed the role of AI within decision-making when it comes to melanoma detection, we constructed the 5 themes. There were 4 main themes and 1 integrative theme (refer to Multimedia Appendix 1 for an outline and additional representative excerpts for thematic context and interpretive validity): theme 1 (integrative theme)—the importance of good clinical judgment; theme 2—AI as just one tool among many within the process of decision-making; theme 3—AI as an adjunct after a clinician’s decision; theme 4—AI as a second opinion for unresolved decisions; theme 5—AI as an expert guide before decision-making.

The integrative theme explained how participants conceptualized arriving at good decisions through the development and display of good clinical judgment; in this context, although AI could be accurate, it often lacked contextual awareness. This was the reference point through which participants discussed their views on incorporating AI. It provided a link to the 4 main themes that described the ways participants then conceptualized AI within the decision-making process when screening for or diagnosing melanoma.

### Theme 1 (Integrative Theme): The Importance of Good Clinical Judgment

This theme provided a link to the other 4 main themes by encapsulating how participants conceptualized arriving at good decisions when detecting melanoma through the development and display of good clinical judgment, “acumen,” or “nous.” This was endorsed as one of the hallmarks of being a good clinician that leads to accurate decisions and optimal patient outcomes. Across the accounts of dermatologists, GPs, and melanographers alike were indicators of clinical astuteness, in particular (1) seeking information from multiple sources, (2) being able to see the broader clinical picture and consider contextual factors, (3) synthesizing and balancing the information that has been gleaned, and (4) applying it accurately to individual patients with their benefit in mind. For example, one participant said the following:

I think putting all these little clues together is part of the point, and each thing gives you a little bit of incremental information when you have to use your judgment, too, of how you weigh bits of information to make a decision. And then you have to also make decisions about how you rate information in terms of what the patient will consider acceptable practice.

ID07, dermatologist

Being a good clinician meant knowing which pieces of information are truly relevant in each specific case and what clinical importance to assign them:

...when you have to manage a patient, you don’t just see a dermoscopy image and say, “okay, it’s melanoma or it’s a dysplastic nevus.” You take into account so many different factors. There are so many variables that we consider when we have to offer a specific management to the patient.

ID01, dermatologist

It was through the practice of repeatedly making decisions for oneself that clinicians developed expert clinical acumen when it comes to melanoma detection, whereas new and inexperienced clinicians tended to “get stuck on one feature” (ID07, dermatologist) and failed to incorporate enough information from multiple sources.

In this context, participants described AI as impressive when arriving at accurate decisions, but the limitations of AI as a “decision-maker” arose because “AI can’t put the lesion into the context of the patient” (ID08, GP). Participants often noted “respect” for accurate AI programs and anticipated considerable improvements in accuracy in the future (“when put under the pressure you can see, wow this AI is fast and pretty accurate! So after that, I had a lot of respect.” [ID12, dermatologist]) while also highlighting that AI is trained to recognize images of lesions and make decisions in a way that is entirely different from the way clinicians use their acumen to make good decisions with real patients. For example, one of the dermatologists said the following:

It’s looking at an image. We’re not looking at image. This is a completely artificial way of looking at our job...We’re looking at a patient who has 100 lesions, and we’re putting so many of their risk factors in the question.

ID17, dermatologist

While an accurate AI might come to a correct decision, often the issue for participants was being aware that AI could have little clinical acumen or “nous” and make errors that would be unusual for an expert. This created a need for clinicians to maintain a kind of vigilance against accepting AI output as though it simply reflects the product of good clinical judgment:

...there’s still a lot of human interaction which goes into clinical decision formulation and management planning, which I think is not yet incorporated in AI appropriately, satisfactorily.

ID07, dermatologist

One highly experienced melanographer summed this up by saying the following:

...that’s something that AI can’t do. It can’t grab that patient, the background, at this point anyway. How long has it been there? Is it coming and going? Were you aware of it? What does it feel like? From an actual physical touch is it rough, is it raised, is it soft, is it squishy. Then from the patient experience is it sore, is it tender, it is itchy?

ID16, melanographer

This impacted the way participants went on to conceptualize and navigate their interactions with AI tools, trust AI’s output, and position AI within their own decision-making process:

The main question is: will the clinicians become better from using the AI or not?

ID03, dermatologist

This conceptualization of good clinical judgment and acumen informed the themes described subsequently.

### Theme 2: AI as Just One Tool Among Many Within the Process of Decision-Making

This theme encapsulated a view of AI as a “tool” for consideration when forming decisions rather than AI as a “decision maker” per se. In this light, viewing AI output as simply one piece of information to be synthesized meant that good decision-making on the part of clinicians required knowing *how* or perhaps *whether* to refer to AI output in the course of reaching a decision:

Every field of medicine, as technology improves, if you don’t use it well then you have to say why not? So this is no different. Just another tool. It’s just a more difficult tool to interpret.

ID13, dermatologist

But crucially, doing this well was largely dependent on clinicians already having well-developed expertise in order to be judicious in how they interpreted AI output as an “incremental” piece of information (“*...*a useful tool for an intelligent doctor who knows its limitations” [ID08, GP]). For example, using an AI tool that detects small changes in a lesion over time still requires the clinician to have the expertise to know which change is clinically important for real patients:

It [AI] detects change, but it detects every little change...and in the end we manually compare their sequential images and we turn off all the AI, because it circles so many things. It’s just a pain.

ID07, dermatologist

As such, these clinicians were careful not to assign more weight to AI output than it deserved:

I never base solely on AI...So, I never say “AI said this, so I’ll do this.” No, never.

ID21, dermatologist

This served to highlight how the development and implementation of expert clinical acumen was an inherently important part of making good decisions when this involved conceptualizing AI as one tool among many:

Look the way I perceive it [AI] would be an incremental step so...but it doesn’t replace taking the history, examining the patient, selecting my lesions, it will give me one incremental piece of information in the specific investigations required.

ID07, dermatologist

By conceptualizing AI as “simply a tool” that may add one piece of information to be synthesized, often the key question for highly experienced clinicians was whether this tool then added any information beyond what they could already glean through their own good clinical acumen or “nous” to assist in the formation of a decision. As such, many experienced participants in this study felt they did not need the information from an AI tool to help inform their decisions:

...de novo interpretation of a particular lesion is where the AI can be helpful, but I don’t believe it helps with someone that already has quite a considerable amount of training in lesions.

ID11, dermatologist

One GP with considerable familiarity with AI described the experience when receiving information from AI tools currently being developed in research settings:

To be honest, I’ve used this for a number of years, and I don’t often find anything new with it...if you’re a really good dermoscopist, what we’re finding with our data is that the GPs are better than the AI here. We’re finding melanomas smaller and earlier than the AI is.

ID02, GP

It is not that participants invariably expected AI would be wrong in any given instance (some AI tools could certainly be accurate), rather their wariness was precisely because they knew AI does not incorporate the entire scope of information that expert human clinicians ordinarily make use of. It was because clinicians could not always anticipate being able to relate to AI on the common ground of good clinical acumen (eg, experience, ability to synthesis a wide range of information, and ask questions) that some spoke about their need to better understand exactly how AI arrived at decisions, so they could anticipate how AI output could be incorporated as one piece of information into their deliberations:

If someone is coming with an AI, I need to understand where to place it, and the training and testing of the data set, so I can see if my things are the same as what they’ve been doing. I need to understand the data, how they’ve been taking their images, how they designed the algorithm, how they’ve been processing their images, if there was some process, and then of course, I need all the metrics. I need the clinical validation. I need to have that in my work flow, with my patients, and so I need to know if it has a good sensitivity and specificity, and all the performance metrics...I need to know the limitations, because there are always limitations.

ID17, dermatologist

### Theme 3: AI as an Adjunct After a Clinician’s Decision

Participants often described the value of AI as a source of reinforcement for the decisions they had already made when screening patients for suspicious lesions or diagnosing melanoma. Participants talked about AI by framing it as an “adjunct” or “auxiliary” when it comes to doing skin checks, inspecting individual lesions or diagnosing melanoma. For instance, “I would still be using my clinical acumen and still would be doing skin checks and using this as an adjunct” (ID09, GP). More specifically, participants across clinical groups explained how they were often inclined to position AI *after* they had engaged in their clinical decision-making because this allowed them to first enact their own good clinical acumen and then use output from AI tools to “validate” this. Importantly, this was even when clinicians felt confident in their decision. For instance, two melanographers described the following:

...mostly I find it confirms, more than changes, what I’m doing...It’s more supporting and confirming.

ID15, melanographer

I make my decision, and I know in myself “I think it’s this.” I pop the AI on and then it’s a nice confirmation. Hypothetically, maybe it’ll catch something I didn’t think of, but if you’ve done the first part right, that shouldn’t be happening.

ID20, melanographer

Participants described how seeking to use AI for confirmation and reassurance after they had made a decision was different from seeking out information from AI to help *form* their initial decision (as seen in theme 2). When formulated as a “checker,” experts were not necessarily seeking out AI’s “opinion” to help them make the actual decision (eg, “Is this lesion suspicious?” “Is this lesion change worrying?” and “Should I biopsy this lesion?”), rather, they were seeking out validation for the decisions they had made. For instance, a melanographer who had used an AI tool for screening lesions said the following:

I definitely like it as a tool to cross-check my work...Often, even not so much to get AI’s opinion. I suppose I use AI to validate and reinforce some of the decisions that I’ve already made. Often, I’ll already have assessed something on the skin.

ID24, melanographer

Indeed, participants from all 3 clinical roles described how they enjoyed the feeling of seeing AI tools confirm their good clinical acumen (“I quite like it when AI agrees with me*”* [ID24])*.* One dermatologist said the following:

Whenever I think something’s benign, AI reassures my decision.

ID21, dermatologist

They felt it reinforced their good clinical acumen and gave them confidence in their decision-making, particularly in the context of feeling anxious about the prospect of failing to detect a melanoma:

If you want me to give you a take home point from my use, it would be just confidence changes, but that’s it.

ID21, dermatologist

This was also the case for melanographers when examining patients with a large number of unusual looking lesions, where AI was described as being like “a reassuring little friend in clinic” (ID20, melanographer). Participants described how one advantage of incorporating AI as a “checker” was that it helped clinicians to be vigilant when assessing patients. AI was not making decisions before or in lieu of the application of a clinician’s own good judgment, rather, when the AI tool “flagged” a lesion, the clinician saw it as an exhortation to ensure they had thoroughly applied their own good clinical judgment in the first place:

If I knew the AI would insist on me looking at it, that would be good.

ID10, dermatologist

It was an invitation to “double check,” confirm their own decision, and be reassured, as described by a dermatologist:

...it makes you more vigilant, which is only a good thing. So long as you manage that balance well, where it doesn’t turn into paranoia.

ID22, dermatologist

### Theme 4: AI as a Second Opinion for Unresolved Decisions

In a small number of cases where highly experienced clinicians were still genuinely unsure about a definitive diagnosis after they had applied their clinical judgment, they described seeking out AI for a “second opinion” (“*...*look in truth, I do look at it quite often if I’m not sure about something, and if I’m vacillating” [ID07, dermatologist]). Participants across all 3 clinical groups likened this specifically to the way they would apply due clinical diligence by seeking out one of their colleagues for a second opinion, which is a familiar practice for clinicians. For instance, one melanographer said the following:

I only use it for things that I’m not sure about. Yeah, that’s probably where my years of experience come in.

ID15, melanographer

Importantly, though, there were varying accounts of whether seeking a second opinion from AI in this way was akin to seeking out a colleague with more expertise, a peer, or perhaps a “less expert colleague.” For some, AI could be a second opinion with great decision-making capacity by virtue of it having access to a large training dataset:

Having the AI is just like having another person in the room. It’s actually swarm intelligence.

ID08, GP

Whereas for others, the current state of AI meant that its opinion was perhaps not as authoritative:

I can tell you, I’ve used the AI and then second guessed myself. I think that’s what it’s there for. It is like having a second opinion from a colleague, if you like, but again, one that’s not validated. One that’s not ready. A less expert colleague, perhaps.

ID22, dermatologist

For some highly experienced clinicians, the fact that their clinical acumen still left them with a degree of uncertainty about a lesion was often reason enough for them to decide on the most cautious course of action (eg, biopsy or excision), regardless of the AI’s second opinion:

I would use that only for an additional opinion you know the same way I use my colleague’s opinion when I am not sure about a lesion of concern. If there’s a discordance between us I think I just do what I feel. I would do the same with AI. If the AI says this is benign, and I still feel that this is not benign, or it is something that must be excised, I would go for the excision.

ID01, dermatologist

Others thought using AI as a second opinion for equivocal lesions may help them prevent biopsy or excision if it allowed them to incorporate some insights that would lead to a more precise diagnosis rather than simply reverting to caution. This might help reduce the removal of benign lesions when it is not needed and be to the patient’s benefit. For instance, one melanographer said the following:

...as long as it’s accurate, as accurate as it can be, I don’t really see any disadvantage of having a second opinion right in front of us. Yeah, I think that it’s more thorough and more accurate if you have the person, myself, and then a system to help you as well.

ID19, melanographer

### Theme 5: AI as an Expert Guide Before Decision-Making

The prospect of patients receiving unbeneficial treatments or melanoma diagnosis being missed was a salient concern:

The worst thing is you have an inexperienced practitioner and they cut stuff out that doesn’t need to be cut out and they’ve actually missed the important thing.

ID04, dermatologist

With this in mind, a number of participants thought that AI output could potentially act as an “expert guide,” for example, when screening for suspicious lesions:

For non-dermatology practitioners, so GPs and other health care specialists, AI tools may improve the identification of suspicious lesions or otherwise the reassurance of lesions that are completely benign.

ID01, dermatologist

But importantly, the prospect of clinicians referring to AI output *before* they had thoroughly engaged in their own decision-making (or in place of a clinician engaging in decision-making) was also predicted to be “to the detriment of the patient” (ID08, GP) if it inclined clinicians to de-emphasize or set aside the need for “good clinical acumen.” It was seen as a “short cut” that could leave clinicians susceptible to error through overreliance on AI and deskilling:

...if you are not as good at seeing melanomas, then AI may help you to do that. But it could easily provide false reassurance because we know it doesn’t always pick them up. And it could lead to reliance on AI and deskilling, and that’s my concern. It’s not a substitute for good clinical acumen.

ID02, GP

At stake was the distinction between using good clinical judgment to *incorporate* information from AI into one’s decision-making (“interpreting” information from AI as part of making one’s decision as seen in theme 2) and actually allowing one’s clinical judgment to be “influenced” by AI in a way that detracts from displaying good clinical acumen. Given that there was widespread recognition that good clinical acumen involved making a considered judgment based on many sources of information, some explained the value of making a conscious effort *not* to use AI as an expert guide. Some participants described the importance of ensuring their initial decision-making was not influenced by AI at all, so that they were not being “told what to do” by AI, but rather retaining autonomy in their decision-making. For example, one GP with considerable experience with AI tools as part of research said the following:

Every patient we reviewed them with a dermatoscope prior to doing any AI analysis...we very, very deliberately take logistical steps [to do that].

ID02, GP

That is, it was important to first form one’s own assessment before attempting to incorporate information from AI as a tool into the decision-making process:

If you click an AI button before you’ve made your assessment, you won’t actually get a real idea what you’re thinking what the lesion was. It’ll actually adversely influence your decision-making process.

ID13, dermatologist

Others similarly cautioned against the temptation to use AI to guide their decision-making (rather than “confirm” or “validate” their decision-making) by likening it to “the ultimate shortcut for people to not learn stuff” (ID12, dermatologist) or “an excuse for not becoming an expert yourself” (ID08, GP). For instance, 3 experienced melanographers noticed the way new trainees were using an AI tool for screening suspicious lesions:

I don’t know that it’s a good idea, in new melanographers. I really think you need to sort of trust your instincts and use your knowledge in the beginning to really know what you’re looking at. We’ve had a couple of new people start with that (AI), and I guess they rely quite heavily on it.

ID15, melanographer

So we have someone here who is newer and she was saying you could definitely, as a new, person doing this rely on AI without having the training, and the experience. And so it’s getting that balance, I suppose, between the experience and not totally relying on it.

ID16, melanographer

I notice the new nurses, they’re using it definitely as their guidance. Whereas I almost use it to just reinforce my decisions. I think at this point I definitely tend to have made my decision up already.

ID24, melanographer

Participants thought that if inexperienced clinicians (whatever their clinical role) used AI tools as a guide to melanoma detection without developing and exercising their own clinical acumen, then although they may at times benefit from AI’s accuracy, they may also be prone to reinforcing errors arising from AI’s lack of acumen. As such, this led some participants to the view that, in the hands of inexperienced clinicians, using AI as a guide was likely to be a mistake, hence perhaps AI tools ought to be considered “expert only” devices:

At the beginning we thought that it will be a tool that will be a significant help for non-experienced users, but for experts, okay, it’s not so important. I’ve started to believe it is the opposite. You have to be quite good in order to be able to deal with the strange decision making that the machine makes, and to understand when you should follow and when you should ignore what the machine is saying. This requires quite a lot of confidence and experience. An inexperienced user is prone to fall in all the traps that might happen when you use these tools.

ID23, dermatologist

The prospect of using AI as part of screening or triage of patients to filter out benign lesions (the task of melanographers) was alluring for a number of dermatologists and GPs who thought this may increase their efficiency. However, many participants were also skeptical that the current AI tools were sufficiently accurate to wholly defer to as an expert guide in this decision-making. Indeed, several melanographers in this study talked specifically about the dangers of inexperienced clinicians overrelying on AI by using it as though it were an “expert guide” for lesion screening. What is instructive is that some melanographers in this study, with access to AI as part of lesion screening, gave credence to this view:

I think I rely on it [AI]. I do. Yeah, I think I relied on it like the most when I first started, but I think as you like learn more and see more skin and see more like diagnoses, I think slowly you start—I don’t rely on it as much anymore, but I definitely still rely on it.

ID19, melanographer

This also lent support to the view that using AI as an expert guide may “deskill” clinicians, by limiting opportunities to enact good clinical acumen. For instance, one melanographer talked about remaining vigilant and not to overrely on AI in a way that might atrophy her good clinical acumen:

What if tomorrow we stopped using it [AI]? Will I lose my clinical skills?... sometimes I think to myself at the end that’s why I stay quite cautious.

ID20, melanographer

This concern was echoed by other participants who foresaw the prospect that positioning AI as a guide to decision-making may invariably impair their good clinical acumen. Again, this is an acknowledgment that AI tools can be potentially useful or helpful but not if they are used in ways that detract from developing good clinical acumen, as described by a dermatologist:

Maybe our human expertise lowers a bit, or gets a bit impaired, that we have the (AI) support. And we’ve trusted so much that we stopped developing ourselves.

ID03, dermatologist

## Discussion

### Principal Findings

A prominent narrative within the dermatology literature sees AI as supporting clinical decisions [6], with advocates pitching the use of AI to “augment,” “assist,” and “aid” the detection of melanoma via a human-computer collaboration [22,23]. For instance, Esteva et al [1] pointed to the potential of AI algorithms for “augmenting clinical decision-making for dermatology specialists”; Brinker et al [3] said that “artificial intelligence algorithms may successfully assist dermatologists with melanoma detection in clinical practice,” and Haenssle et al [2] said that AI tools may “aid physicians in melanoma detection.” However, the notions of augmentation, collaboration, support, and assistance may entail a very wide range of actual functions for AI in the context of decision-making. We find that end users conceptualize these terms in an array of potentially disparate ways that impact AI’s incorporation into clinical workflows. These meanings are important for clinical groups, AI developers, and policy makers to understand so that the development of clinical workflows and guidelines for AI use are appropriate and acceptable to end users (in this case, end users being dermatologists, GPs, and melanographers). Indeed, the potential prospects of AI improving decision-making have been tempered more recently by concerns that algorithms with superior performance to human clinicians in research settings do not necessarily translate into better performance with actual patients in the context of real-world decision-making [17,24]. AI algorithms for assessing malignant lesions can often perform less well outside experimental settings [25]. Nevertheless, some AI tools have already been approved for screening, and some skin imaging platforms allow clinicians to access diagnostic AI assessment of lesion images with the caveat that these AI tools have not yet been validated or approved for clinical decision-making.

This study brings to light a timely perspective on how clinicians involved in melanoma detection conceptualize the use of AI within their decision-making processes and explains how they view the role of AI within the context of good decision-making. Our findings can be further contextualized by considering previous assessments of clinician attitudes toward AI in dermatology. The existing literature has been largely characterized by brief surveys exploring broad impressions about the potential impact of AI, and several of those studies have highlighted optimism among clinicians about the prospect that AI may improve melanoma screening [12] and the accuracy of decisions. Surveys from European and Middle Eastern countries as well as China, the United States, and Australia [8-11,13,14,22] commonly reveal expectations that AI-supported decision-making will be beneficial to the field [8,9,11,12], with many of those surveyed seeing AI as having the potential to improve diagnostic accuracy or other decisions [9,10]. Among the biggest expected benefits is the potential for AI to improve patient access to melanoma screening, although inaccurate AI screening or diagnosis is a major concern [12]. In focus groups with Dutch dermatologists [16], greater diagnostic accuracy was cited as the leading perceived benefit of AI potentially leading to “fewer missed skin cancer diagnoses and less unnecessary biopsies and excisions of benign skin lesions.” However, dermatologists also held concerns about the use of AI tools if their accuracy with real patients fell short of the current abilities of expert clinicians.

The use of qualitative methods in our study has extended these snapshots by showing how these beliefs need to be interpreted in line with the way AI is conceptualized by clinicians within the decision-making process, particularly those who have already used AI in some way. Our findings suggest that the potential benefits of AI for improving accuracy in diagnosis or screening depend on where in the clinical decision-making process AI is used, how clinicians engage with it (eg, as a tool, checker, second opinion, or guide), and the level of expertise and experience of the clinicians using it. As such, while clinicians may have an overall view that AI can “improve accuracy,” the extent to which they endorse a specific workflow that includes AI is likely to be contingent upon these kinds of details.

Formulating workflows that are sensitive to the positioning of AI has been identified as a critical part of using AI to enhance clinical decisions [26], and our themes could be used in future studies as touchpoints to assess clinician endorsement of various AI workflows. Importantly, our findings show that the importance of these concepts extends beyond dermatologists—they are seen in the accounts of GPs as well as melanographers—and given that melanoma detection is often a multilayered process for patients that encompasses interactions with several clinician groups, it is important for future research to recognize this.

Our main themes support previous evidence that the “confidence” clinicians have in their decisions may be improved with the use of AI. For example, in their 2020 survey with dermatologists from Australia and New Zealand, Scheetz et al [12] found that “improved diagnostic confidence” was one of the most cited potential benefits of AI. However, our study also shows that, for many clinicians, this was the result of using AI as a “validator” for their decisions or as a reassuring adjunct (see theme 3), rather than using AI to help them make the decision in the first place. Similarly, while clinicians see the potential for AI to improve diagnostic accuracy, our results add nuance by showing that many experts conceptualize this as applicable only to less experienced clinicians and mainly when AI is used as simply one “tool” among many or as a “checker” rather than deferred to. This fits with other findings [12] showing that when detecting skin cancer in experimental conditions, experienced clinicians largely ignored AI output if they were confident of their decisions, whereas inexperienced clinicians were more likely to accept AI-output that contradicted their initial decision, so when the accuracy of the AI tool was of a lesser quality, this put decision makers at risk of error.

This understanding of how AI is conceptualized by clinicians allows a better interpretation of clinician views on the acceptability of AI and human-computer collaboration. In determining what the optimal clinician-computer collaboration should look like, it has been pointed out that “the ideal positioning of AI in relation to the clinician also needs to be considered” [26]. Our study shows how participants across clinical roles commonly articulated a major conundrum about the positioning of AI for melanoma detection that encapsulates a number of the potential benefits and drawbacks associated with each conceptualization of AI; in trying to accurately detect melanoma, although AI may be of benefit to inexperienced clinicians when used as an “expert guide” before they engage in their own decision-making, the potential for this to lead to overreliance, deskilling, and a failure to recognize AI errors when they occur may mean that only expert clinicians have the required acumen to use AI properly as “one tool among many” to inform initial decisions. That is, when conceptualized this way, only those with already well-developed clinical judgment are thought to be able to appropriately engage with the limitations of AI (the “traps,” as one participant put it), including AI’s lack of contextual awareness. However, at the same time, experts in this study often described how they did not necessarily see a strong need for AI to inform their initial decision-making; instead, they preferred to be able to rely on their own clinical judgment when making decisions.

In this study, while some participants conceptualized AI as offering “one piece of information” to be judiciously interpreted within their synthesis, they often did not see any extra information being gleaned from current AI beyond what they could establish about patients through their own good clinical judgment, and they could be wary of allowing AI output to “influence” their initial deliberations in ways that undermined their independence as a decision maker. However, this perhaps raises queries about the extent to which experienced clinicians then truly seek to “collaborate” with AI on decisions or use it to support their decisions in ways that may actually improve accuracy. Participants often thought that AI’s main benefit was in reassuring their own decisions after they had been reached, as a kind of “checker,” “validator,” “confirmation tool,” or in a small number of equivocal cases as a genuine “second opinion.” As such, this perhaps raises doubts about the extent to which experienced end users always see AI as having the potential to “support” or “assist” within their decision-making process in beneficial ways, again highlighting the importance of understanding what clinicians mean in this regard. Further evidence will be needed to elucidate whether this way of positioning AI in the process will maintain benefits, such as clinician autonomy, without resulting in drawbacks, such as failure to make best use of an accurate AI. Indeed, one recent study of AI use among dermatologists found that despite high confidence in the AI tool, many opted to continue relying on their own decision-making [22], and there is evidence that people often “ignore (AI) recommendations because they do not trust them; or perhaps even worse, follow them blindly, even when the recommendations are wrong” [27]. Our findings extend those from some experimental conditions and surveys showing that experienced clinicians largely ignore AI output when diagnosing melanoma if they are confident of their decisions, whereas inexperienced clinicians are more likely to accept AI-output even when the accuracy of the AI tool was of a lesser quality, thus putting them at risk of error [7]. Still, Tschandl et al [7] found that “faulty AI can mislead the entire spectrum of clinicians, including experts.”

With the positioning of AI being very important to the way AI is conceptualized, it will be pertinent to consider practical matters, such as whether clinicians can elect when or whether they see AI output and under what circumstances. Similar challenges in knowing exactly how to incorporate AI in ways that promote good diagnostic decision-making have also been reported by clinicians using AI to detect other types of cancer (eg, radiologists using AI to detect breast and lung cancer) [28]. Interpreting our results through the lens of commonly applied frameworks for assessing user acceptance of technology may yield insights into our participants’ perspectives. A recent review [29] found that widely used frameworks such as the technology acceptance model [30,31] and the unified theory of acceptance and use of technology [32] include key factors such as performance expectancy or perceived usefulness among the strongest predictors of behavioral intentions. Regarding our results, constructs such as performance expectancy or perceived usefulness conceivably encompass a range of views expressed by our participants, including (1) the current accuracy, sensitivity, or specificity of AI for melanoma detection, (2) the extent to which AI takes into account the broader patient context, and (3) the perceived need (or lack thereof) for experienced clinicians to rely on information from AI rather than their own clinical discernment, to make good decisions when screening for suspicious lesions, detecting change, or arriving at a diagnosis.

Across our themes, participants described how the performance or perceived usefulness of AI as part of melanoma detection may vary depending on matters such as the kind of role it may play in the decision-making process, the position it occupied in the workflow, or the relative expertise of the clinician. Nevertheless, it is worth noting that the rapid development and unique nature of AI technology has tested the ability of many older technology acceptance models to confidently predict behavior about AI. This is particularly the case in health care settings where there is a complex interplay among social, technical, and organizational structures and with many stakeholders [33]. As such, it is important for inductive work to reveal how AI use within specific cases is conceptualized by stakeholders and what meanings they attach to AI within the scope of their existing values and obligations.

The way participants in this study have conceptualized the role of AI within good decision-making also points to important ethical considerations. The use of AI within dermatology (and indeed health care more broadly) raises many already well-described ethical issues related to data privacy and ownership, transparency, and equitable access [34]. Navigating these issues in an optimal way is likely to require considerable assessment. Our study suggests that ethical obligations to act in accordance with AI may be placed upon clinicians when seeking and receiving information from AI. These may then impact the extent to which they feel accountable for decisions. For instance, if clinicians are to act in their patients’ best interests, then it is reasonable to expect that they ought to rely on the best available information when making decisions, that is, there is a prima facie moral obligation within clinical encounters to treat patients based on the best or most accurate available information (at least, it would be unethical for clinicians to *prefer* to rely on information they know is from an inferior or less accurate source). When AI is conceptualized as an “expert guide” then, it is implicitly installed as a kind of epistemic authority in relation to the clinician (AI is the “expert”). This may then create an ethical directive for clinicians to act upon the advice of AI accordingly, because if AI is the acknowledged expert, then it seems hard to justify ignoring its output or making contrary recommendations. However, this also appears to position AI as the accountable party in the workflow. Throughout the study, participants also strongly endorsed a seemingly countervailing imperative for clinicians to “understand when you should follow and when you should ignore what the machine is saying” (ID23), that is, this appears to be a directive to be judicious in accepting AI’s decision. In this study, when participants talked about approaching AI output in a judicious rather than deferential way, it appeared to be borne out of two views. First, the current AI tools were not sufficiently accurate to justify an obligation to follow their output without engaging in independent decision-making. second, clinicians are ultimately accountable for their decisions, meaning that “deferring to AI” could be tantamount to recusing oneself from a core ethical responsibility as a clinician (and to patients). This may be why conceptualizing and positioning AI as a “checker” or “second opinion” (themes 3 and 4) was more readily endorsed by many participants; doing so may be seen to preserve their ability to act as the epistemic authority, enact their moral responsibility to promote the welfare of patients, and potentially negate any potential ethical obligation about following the AI. For instance, participants seemed more comfortable about dismissing or “overruling” AI when it was simply consulted as a checker or second opinion. However, the extent to which this is justifiable, or will hold in all potential situations, is unclear. When the accuracy of the AI is known to exceed that of the clinician, then this ability may be left in a perilous ethical state (although it is also important to recognize evidence that AI accuracy in experimental settings is typically far superior to “real-world” AI accuracy [25]). A deeper examination of the scope of ethical obligations raised by AI within specific melanoma screening workflows is certainly warranted to understand how to best implement any future proposals for widespread screening programs.

In doing so, it is worth noting that patient or consumer views toward AI in melanoma detection (or at least, what clinicians believe to be the views of patients) may also in turn impact how clinicians adopt and use AI. Several recent studies have found that most dermatology patients report having few, if any, concerns about AI being used by specialists to diagnose skin cancer as long as diagnostic decisions are not made by AI alone [35], with diagnostic accuracy and explainability as being features of AI that are most important [35-37]. Given that the mere presence of AI within decision-making workflows imbues AI with at least some epistemic legitimacy, this may raise questions for patients about the extent to which AI ought to be deferred to (eg, be used as an expert guide and as second opinion), which may, in turn, impact the extent to which clinicians act accordingly to maintain their patient’s trust. Different conceptualizations of the role of AI may present different ways of dealing with issues such as clinician-AI discordance. These issues are likely to be made starkly apparent in the implementation phase, for instance, in situations where AI output is available to patients and clinicians in real time. It would be pertinent for implementation scientists working on the development of melanoma detection workflows to consider how the different conceptualizations of the role of AI described in this study may accord with the views of patients, and, in turn, impact their trust and acceptance of AI in the process.

The possibility of AI inducing deskilling through overreliance on AI has often been identified in the literature [38-40]. While this is a recurring concern for health care practitioners [39], the potential for AI to hinder learning or erode already-acquired competency permeates more broadly [40]. This study showed that experienced clinicians involved in the detection of melanoma were cognizant of this potential. Some described how they tried to ensure they adopted decision-making workflows that resisted this, for example, by only using AI after they had made an initial decision. Importantly, this study also showed that the prospect of reliance on AI was not merely hypothetical; our interviews uncovered evidence that some newer melanographers who had been trained to use AI to identify suspicious lesions (and refer them for dermatological review) were aware that their anxiety about missing potential melanomas inclined them to regularly rely on AI output as an expert guide, given their initially limited clinical experience. More experienced melanographers were wary of doing this and held concerns that overreliance on AI would facilitate them to “lose their skills” or clinical judgment. This was concerning for participants given that the development and display of good clinical acumen reflected what it meant to be a good clinician; it entailed making decisions in a way that takes into account many pieces of information from the patient, learning how to balance potentially relevant clinical information through experience and reflection, and seeing the broader context of the patient with their interests in mind (refer to the study by Tsang et al [41] for a similar view). Due to this, participants described a primarily AI-led decision-making model for detecting melanoma as one with the potential to stifle the development of good clinical judgment among junior and inexperienced clinicians and atrophy the skills of already experienced operators if it led to good acumen being too regularly bypassed in favor of efficiency. With this in mind, it may be beneficial to investigate how the development of training programs around the use of AI in dermatology as well as clinical guidelines on AI use may take our findings into account. In recent years, clinical groups have published position statements designed to inform dermatologists on the appropriate use of AI. For example, the Australasian College of Dermatologists has outlined out a number of recommendations for AI adopters designed to address commonly seen issues in the application of AI in medical settings (eg, privacy, a desire for transparency in output and training data, and the need for evidence of accuracy and validity) [42]. They recommend dermatologists develop basic knowledge and skills in the use of AI, such as “appropriate use,” understand that “output from AI models can produce false-positive and false-negative results,” and that their “decision making may be biased by using AI.” These very broad-level recommendations could be extended by considering how the different conceptualizations of AI described in this study reveal what clinicians mean by good decision-making in the context of AI use and what our findings indicate, for example, about the concern clinicians have regarding overreliance, deskilling, and maintaining good clinical acumen. In discussing these issues, rather than referring to broad notions of “AI support,” it may be more useful to construct more specific recommendations by referring to the constructs we describe here, such as “AI as a tool within decision making,” “AI as a checker after decision making or second-opinion on equivocal cases,” and “AI used as an expert guide before decision making.” This may also improve guidelines on use so that decision-making workflows are sensitive to the desire of clinicians to retain the ability to exercise and develop independent decision-making skills while using AI and also take into account the whole clinical context of the patient.

Given the rapidly evolving nature of AI technologies within dermatology and the health care space more broadly, we suggest several other areas for future research in light of our findings. First, there is a need to evaluate the real-world effects of clinical workflows that position AI in the decision-making process in ways resembling those outlined by participants in this study (eg, “checker,” “second opinion,” and “expert guide”). For instance, designing and implementing optimal melanoma screening programs will require good validation studies of how human-AI interactions are affected when AI is variously positioned before, after, or during human clinical inspection. Second, it is yet to be determined how the conceptualizations of AI described in this study may translate across melanoma detection workflows that use a range of imaging technologies. For instance, the question arises whether the potential role of AI in good decision-making differs when operating as part of 2D dermoscopic imaging platforms as opposed to 3D total body photography for melanoma screening. The design parameters of some imaging technologies may determine the extent to which some of the AI roles described here are able to be operationalized and how this affects decision-making needs to be better understood. Third, experimental work can provide evidence for how the positioning of AI may impact potential deskilling of experts or possible delayed skill acquisition of novices. This kind of experimental work may also yield insights for developing effective ways to allow a human-AI feedback loop to occur in real-time decision-making as a way of increasing the explainability of decisions. Fourth, there is considerable scope for further qualitative and quantitative research to better understand how the conceptualizations of clinicians described in this study accord with the views of consumers, particularly patients with high risk of melanoma who are likely to be a priority population for melanoma screening and lesion monitoring.

### Limitations

While there was diversity in experiences in practice settings among interviewees, overall integration of AI into everyday clinical use remains uncommon. Most of the sample (n=25, 83%) in this study reported having some experience with AI tools for melanoma detection, but currently no AI tool for the diagnosis of melanoma based on dermoscopic images has been approved. Therefore, views on AI for this purpose are based on individual field experience with unapproved tools, testing, or reader studies. Practical experience, testing, and comparison of various tools in a clinical setting would likely provide additional insights not captured in this study. AI tools are constantly evolving, and some views may be based on early AI tools that are still in development (eg, those with experience through research). Our purposive recruitment was done so that we could elicit, where possible, reflections on the way participants may have already interacted with AI tools when making decisions rather than only form views about hypothetical situations. This is a strength of the study as participants were not reliant on speculating about hypothetical situations; however, we do also acknowledge that the experience of the sample may not translate to all clinicians, particularly those in other health care settings or cultures.

Our sample predominantly comprised clinicians from Australia and New Zealand, raising the question of generalizability of the findings to broader health care systems and cultures. Australia and New Zealand have the highest rates of skin cancer worldwide [18], with populations (eg, in Queensland) having very high rates of sun exposure. Australia has implemented a decades-long public health campaign devoted to fostering sun-protective behaviors. While there is no coordinated widespread melanoma screening program, a shared public-private health care funding model means there is a very wide coverage of consumers seeking screening through opportunistic skin checks conducted by dermatologists, skin cancer clinics, GPs (including those with a special interest in skin cancer), and other clinicians such as melanographers. As such, clinicians working in melanoma detection in Australia and New Zealand (as well as general practice clinicians not specializing in skin cancer) are highly familiar with examining many consumers with highly sun-damaged skin and see many types of skin cancer. This familiarity may mean that a high level of importance is placed on good clinical acumen when it comes to melanoma detection, and they perhaps feel less inclined to rely on AI. The use of AI as an expert guide may be more acceptable to clinicians in other health care systems with less experienced clinicians, lower rates of melanoma, or where consumers have less access to health care.

Research related to AI-clinician collaborations for melanoma detection has, to date, understandably often focused on the decision-making of dermatologists; therefore, a particular strength of this study is the elicitation of views on AI from GPs and melanographers (in addition to dermatologists), given that they too conduct skin checks, identify lesion change, or make diagnostic decisions (GPs). Notably, there was considerable shared meaning across groups, likely due to the shared understanding of the clinical decision-making process and what entailed “good” decision-making. However, further research is needed to draw out potential differences across these groups, particularly in relation to the use of specific AI tools that are developed for use in practice.

### Conclusions

Clinicians described their conceptualizations of AI in melanoma detection in ways that prioritize the conservation of good clinical acumen, and this must be a priority when developing and adopting AI into the decision-making process. This has implications for who is likely to be the most appropriate user of AI given its limited contextual awareness, and careful consideration must therefore be given to how (and if) AI is adopted in the clinical setting once AI tools are formally approved by the respective authorities. Our study implores a more focused engagement with users about the precise way, and in what position, they envisage AI being incorporated into their decision-making process for melanoma detection.

## Appendix

Multimedia Appendix 1 Outline of themes and additional data excerpts.

## Abbreviations

AI: artificial intelligence
DERM: Deep Ensemble for Recognition of Malignancy
GP: general practitioner
