# Supplementary material for: Exploring the Views of Dermatologists, General Practitioners, and Melanographers on the Use of AI Tools in the Context of Good Decision-Making When Detecting Melanoma: Qualitative Interview Study
Source: JMIR Dermatol. 2025 Mar 24;8:e63923. doi: 10.2196/63923 (PMC11976179; doi:10.2196/63923)
Supplement: Multimedia Appendix 1 [file derma_v8i1e63923_app1.docx]

**Table S1.** Theme outline and additional data excerpts.

| Theme | Description | Additional representative excerpts |
| --- | --- | --- |
| Theme 1 (integrative theme): the importance of good clinical judgment | This entails a clinician arriving at an accurate decision by being able to see the broader picture, combine information, and apply it to individual patients for their benefit. It is developed through experience and repeatedly making one’s own decisions. AI^a^ can be accurate but lack contextual awareness and acumen. | - “You can’t treat a patient in isolation, or a lesion in isolation from the rest of the patient…” [ID05, general practitioner] - “Using your clinical judgment. Because that’s the thing, AI does n’t take into account any of the clinical context of the patient and what you’re seeing.” [ID16, melanographer] - “It’s a multifactorial process. You can never base your decision on a single factor, always you take into consideration many things.” [ID23, dermatologist] |
| Theme 2: AI as one tool among many within decision-making | AI conceptualized as simply one incremental piece of data within the entire scope of information about the individual patient. Knowing how or whether to incorporate AI output judiciously within the decision-making process may aid accuracy, although experienced clinicians may not glean extra benefit. | - “It’s just an additional checkpoint, if you like, that sometimes we take into account...for real decision making with patients today, then the role is minimal. It’s just an additional auxiliary tool that we take a look at.” [ID23, dermatologist] - “An expert clinician doesn’t need AI...I have spent like twenty years doing dermoscopy and been training in it for years. So I am as good as any AI. Possibly not as fast. And I have other bits of information to help me like a patient tells me it’s changed, or they have a concern about it, or it’s different. So I don’t need AI to tell me things right now.” [ID12, dermatologist] |
| Theme 3: AI as an adjunct after a clinician’s decision | This conceptualized AI as a method to reinforce decisions after clinicians have already made them. Rather than clinicians seeking to incorporate AI output directly into their decision, they conceptualized it as a way to “double-check,” “validate,” or “confirm” decisions and provide reassurance of their accuracy. | - “I don’t get a surprise by the answer. You know, more often than not it [AI] goes along with what I am thinking. So maybe that sounds really, really cocky. But I’d say it’s the truth.” [ID15, melanographer] - “I think I use it as a double check. Most of the time it’s nothing, I think it’s very sensitive, not very specific. And I that makes sense. I think I like it that way because I could be very sure that I’m adding specificity and the computer is helping with sensitivity, right?” [ID21, dermatologist] |
| Theme 4: AI as a second opinion for unresolved decisions | When even highly experienced clinicians were confronted with a lesion that they felt genuinely uncertain about, they likened their inclination to arrive at a definitive position by seeking out AI as a second opinion in the same way they would seek out one of their expert colleagues. | - “If you’re any good, you know what’s benign. And then there’s a whole bunch of stuff that is obviously a problem. And then there’s some stuff in the middle that you look at and you go ‘Y’know, I’m not so sure about that.’ And if you’re not so sure about it then you either put the AI on it, if you’ve got the AI, or you just say to the patient, ‘I’m not too sure about this, so if in doubt let’s cut it out.’ I mean, that’s the broad reality. You’ve got those three buckets.” [ID05, general practitioner] |
| Theme 5: AI as an expert guide before decision-making | Using AI as an “expert guide” before a clinician had engaged in their own decision-making. was done to arrive at accurate or efficient decisions without the need for clinicians to rely on their own well-developed clinical judgment but could induce overreliance and deskilling. | “You could definitely, as a new person doing this, rely on AI without having the training, and the experience. And so it’s getting that balance between the experience and not totally relying on it.” [ID16, melanographer]   - “I feel like if I don’t stay cautious I am a bit worried that I’m going to get into a false sense of security with it. And you don’t want to be getting to a point where you’re relying on the A.I. You really want to have your clinical skills being your reassurance and your experience.” [ID20, melanographer] |

^a^AI: artificial intelligence.
